# Decline in the Incidence and Prevalence of Dementia Diagnoses in German Primary and Specialist Care: Lower Risk or Reduced Attention of Physicians?

**DOI:** 10.1002/alz70860_100830

**Published:** 2025-12-23

**Authors:** Bernhard Michalowsky, Wolfgang Hoffmann, Steffi G. Riedel‐Heller, Stefan Teipel, Manas Akmatov, Jens Bohlken, Jakob Holstiege

**Affiliations:** ^1^ Deutsches Zentrum für Neurodegenerative Erkrankungen e. V. (DZNE), site Rostock / Greifswald, Greifswald, Mecklenburg‐Vorpommern, Germany; ^2^ Deutsches Zentrum für Neurodegenerative Erkrankungen e.V. (DZNE) and Institute for Community Medicine / University of Greifswald, Greifswald, Mecklenburg Western Pomerania, Germany; ^3^ Institute for Community Medicine, University Medicine Greifswald, Greifswald, Mecklenburg‐Vorpommern, Germany; ^4^ Institute of Social Medicine, Occupational Health and Public Health (ISAP), Medical Faculty, University of Leipzig, Leipzig, Saxony, Germany; ^5^ German Center for Neurodegenerative Diseases, Rostock, Germany; ^6^ Klinik und Poliklinik für Psychiatrie und Psychotherapie, University of Rostock, Rostock, Germany; ^7^ Zentralinstitut für die kassenärztliche Versorgung in der Bundesrepublik Deutschland, Berlin, Germany; ^8^ Medical specialist for Neurology, Berlin, Germany

## Abstract

**Background:**

A worldwide increase in dementia cases is expected due to demographic changes and the growing elderly population. However, recent studies suggest a declining incidence. This analysis aimed to examine the trends in the incidence and prevalence of diagnosed dementia in recent years based on data from all individuals covered by statutory health insurance in Germany.

**Method:**

This study was based on claim data of primary and specialist care practices (German population coverage: 88%) from 2015 to 2022. Individuals aged ≥65 years with a confirmed dementia diagnosis in at least two out of four consecutive quarters were included. The incidence and prevalence of dementia were age‐ and sex‐standardized calculated.

**Result:**

The incidence decreased by 26%, from 2,020 per 100,000 insured individuals in 2015 to 1,500 per 100,000 insured individuals in 2022. The prevalence decreased by 18% during the same period, from 10,380 to 8,470 per 100,000 insured individuals. These trends were more pronounced in younger age groups and among women. The number of diagnosed dementia cases declined from 1.56 million in 2015 to 1.43 million in 2022, representing an 8.4% reduction.

**Conclusion:**

Despite ongoing demographic changes, German primary and specialist care practices show a significant decline in the incidence and prevalence of diagnosed dementia cases, contrary to expected projections. It remains an open research question whether a healthier lifestyle, better education, and improved management of risk factors by individuals or a reduced focus on dementia diagnoses due to limited therapeutic options by physicians have contributed to these trends. Further analyses of long‐term cohort studies are needed to validate these findings.

